# The Role of *Sox* Genes in Lung Morphogenesis and Cancer

**DOI:** 10.3390/ijms131215767

**Published:** 2012-11-26

**Authors:** Yongzhao Zhu, Yong Li, Jun Wei, Xiaoming Liu

**Affiliations:** 1 Key Laboratory of the Ministry of Education for Conservation and Utilization of Special Biological Resources in Western China, College of Life science, Ningxia University, Yinchuan 750021, China; E-Mails: btxnxu05@163.com (Y.Z.); liyong7732@126.com (Y.L.); 2 Institute of Stem Cell Research, General Hospital of Ningxia Medical University, Yinchuan 750004, China

**Keywords:** *Sox*, lung cancer, lung morphogenesis, transcriptional factors

## Abstract

The human lung consists of multiple cell types derived from early embryonic compartments. The morphogenesis of the lung, as well as the injury repair of the adult lung, is tightly controlled by a network of signaling pathways with key transcriptional factors. Lung cancer is the third most cancer-related death in the world, which may be developed due to the failure of regulating the signaling pathways. Sox (sex-determining region Y (*Sry*) box-containing) family transcriptional factors have emerged as potent modulators in embryonic development, stem cells maintenance, tissue homeostasis, and cancerogenesis in multiple processes. Recent studies demonstrated that the members of the *Sox* gene family played important roles in the development and maintenance of lung and development of lung cancer. In this context, we summarize our current understanding of the role of Sox family transcriptional factors in the morphogenesis of lung, their oncogenic potential in lung cancer, and their potential impact in the diagnosis, prognosis, and targeted therapy of lung cancer.

## 1. Introduction

Lung cancer is an aggressive cancer type and is the third most common cancer in the world. As a leading cause of cancer-associated mortality, it causes over 160,000 deaths annually in the United States alone [1]. Based on its clinical, histological, molecular, and/or neuro-endocrinological characteristics, lung cancer was classified into two main types: non-small cell lung cancer (NSCLC) and small cell lung cancer (SCLC). NSCLC was further subdivided into large-cell carcinoma (including large-cell neuroendocrine lung cancers), bronchoalveolar lung cancer, adenocarcinoma (AC), squamous cell carcinoma (SCC), and mixed histologic types (e.g., adenosquamous carcinoma) [1,2]; this represents 80%–85% of lung cancer cases, while SCLC represents the remaining 15%–20% cases [1,2]. Owing to its high rate of occurrence with a very poor prognosis relative to other cancer types, lung cancer has recently received increasing public attention.

A large body of cellular signaling pathways has been demonstrated to be involved in the cancerogenesis of various types of cancer, including lung cancer, and some of the signaling molecules are targets for the development of drugs in lung cancer therapy [3]. The vascular endothelial growth factor (VEGF) and epidermal growth factor receptor (EGFR) are of two molecules extensively studied for developing novel agents in the targeted therapy of lung cancer [4]. Therefore, understanding the signaling pathways and identifying the key regulators in both lung development and cancerogenesis may provide an insight into the molecular mechanisms of lung cancerogenesis and create new targets for treatment.

Increasing lines of evidence has shown that lung morphogenesis is controlled by many signaling pathways and transcriptional factors, such as the Wnt signaling pathway, growth factors, and transcriptional factors including: the T-cell factor (TCF) and sex-determining region Y (*Sry*) box-containing factor (So*x*) [5]. These signaling molecules have also been discovered to share diverse roles in lung cancer, despite the fact that the cancerogenesis of lung is a multiple progress with more tendency to be influenced through oncogenes, tumor suppressor genes, key signal transduction cascades, microRNA (miRNA), as well as genetic mutation such as gene amplification and epigenetic modification [6–9]. The *Sox* genes encode a family of high-mobility groups that are a family of transcriptional factors and have emerged as potent modulators involved in orchestrating embryonic development and cell fate, organogenesis, stem cells maintenance, and cancerogenesis in multiple processes. The presented review aims to summarize the roles of Sox transcriptional factors in the morphogenesis and malignancy of the lung.

## 2. Sox Transcriptional Factors

With the discovery of the first *Sox* gene (sex-determining region Y, *Sry*) at the end of 20th century, over twenty different *Sox* genes have been identified in mammalians [10,11]. They all share a noncanonical 79 amino acid DNA-binding domain known as the high mobility group (HMG) box domain. On basis of the sequence comparison of the HMG box domain, *Sox* genes are currently classified into eight groups (Sox A–H); Sox proteins with the same group share a high level of identity both within and outside of the HMG box domain, whereas proteins from different groups share partial identities [12]. As transcriptional factors, Sox proteins gain the ability to bind DNA through recognizing of a consensus hexameric core sequence 5′-(A/T)(A/T)CAA(A/T)-3′, although the consensus sequence is not the only factor that influences the DNA binding of Sox proteins [13–15]. Unlike most other transcriptional factors, the members of Sox family prefer to bind to the minor groove of DNA by which the local chromatin space structure will be changed; this may provide a mechanism of enhanceosome formation. Owing to their lower affinity of DNA binding, Sox proteins are widely believed to team up with other transcriptional factors, such as POU (Oct3/4), zinc finger proteins, basic helix–loop–helix and leucine zipper proteins for functioning properly [16–18]. Increased direct and circumstantial evidence has proved that Sox proteins played a crucial role in multiple development process, including embryonic development, disease, and cancer [19,20]. In this process, they were capable of regulating target genes expression as transcriptional activators or repressors depend on both of the cellular and target gene contexts [21]. Furthermore, the activity of Sox proteins could also be modulated during the processes of phosphorylation, sumoylation and/or ubiquitination [22,23].

## 3. *Sox* Gene and Lung Morphogenesis

Lung originates initially from the foregut of early embryo and subsequently differentiates into multiple lineages of lung cell types through sequential periods of pseudoglandular, canalicular, terminal saccular, and alveolar stages. Several signaling pathways, as well as transcriptional factors, growth factors, extracellular matrix, and miRNA have been identified to contribute to the development and maturation of lung [5]. To date, at least four members from *Sox* gene family are known to be involved in lung organogenesis, they are *Sox2*, *Sox9*, *Sox11* and *Sox17* genes [5,24–29].

Sox2 is expressed from foregut to mature lung in a cyclic manner except for alveolus, which plays a crucial role in proliferation and differentiation of respiratory epithelial, trachea, airway branching, and Clara cells [5,28,30,31]. Previous studies in transgenic mice that express a doxycycline inducible Sox2 in the airway epithelium, demonstrated that Sox2 played a critical role in branching morphogenesis of the bronchial tree and the differentiation of airway epithelium [30]. In addition, selectively deleted *Sox2* gene in Clara cells by Clara cell secretory protein (CCSP) promoter-derived Cre recombinase, led the progressive loss of ciliated, Clara and goblet cells in bronchiolar epithelium after the birth of an animal. This also caused a loss of the ability of goblet cell differentiation and mucus production in response to allergen in the respiratory epithelium [31]. A recent study performed in the same group, which utilized a mouse model, demonstrated that Sox2 was conditionally expressed in a subset of respiratory cells under the CCSP promoter, and that Sox2 was capable of promoting the proliferation of nonciliated airway epithelial cells, accompanied with increased expression of cell cycle genes FoxM1, Cyclin A2, Cyclin B2 and Cyclin D1 in mice [28]. Therefore, defining the CCSP-Cre-derived cell population may facilitate our understanding of the roles of Sox2 in the morphogenesis of lung, and the pathogenesis of lung diseases. A subset of reprogrammed alveolar epithelial cells, which consequentially produces hyperplastic lesions with markers of conducting airway epithelium, was also observed in the above study, despite the fact that Sox2 alone was insufficient to transform epithelial cells into tumors [28]. In addition to the indispensable roles in the normal development of lung buds and the differentiation of both tracheal mesenchyme and epithelium, Sox2 has been demonstrated to be associated with the epithelial cell proliferation and repair of tracheal epithelium following the injury [32].

Highly expressed Sox9 was found throughout lung morphogenesis as a downstream gene of Sonic Hedgehog (Shh) modulated by bone morphogenesis protein 4 (Bmp4) and Noggin. These are required for formation and patterning of tracheal cartilage by a mechanism of fibroblast growth factor 18 (FGF18) mediated controlling of Sox9 expression level and pattern [26,33]. Nevertheless, the specific inactivation of Sox9 in respiratory epithelial cells could not alter lung structure, postnatal survival, or repair oxygen injury, thus indicating that Sox9 might not play essential role in the respiratory epithelial cells [34].

*Sox11* gene has been suggested to be involved in a wide range of organogenesis and has a key function in tissue remodeling including the lung. Mice deficient in Sox11 immediately die after birth caused by the significant hypoplasia of lung and other tissue defects [29].

Sox17 is one of the most extensively studied Sox transcriptional factors in the lung development, which was found to be expressed in respiratory epithelial cells of the fetal lung at embryonic day 18 and is restricted primarily to ciliated cell in the postnatal and adult lung [25,27,35,36]. Of note, however, Sox17 mRNA has not been detected in the lung epithelium [27], and a mouse model ectopically expressing Sox17 in lung epithelium also showed that the expression of Sox17 was not in the epithelium but restricted to the pulmonary mesenchyme of the lung (especially the endothelium of the developing lung) [25]. Sox17 is required for the formation of early endoderm, activates the cell cycle, and reinitiates multipotent progenitor cell behavior in mature lung cells, as well as other development processes such as cardiovascular development, fetal hematopoietic stem cell maintenance, and angiogenesis [25,35,37–40]. Conditional expression of Sox17 in mature respiratory epithelial cells of mice showed formation of hyperplastic clusters of cells and respecification of alveolar progenitor cells toward proximal airway lineages [25,27]. However, ectopic expression of Sox17 in the epithelial cells of the mouse embryonic lung led to inhibiting peripheral epithelial cell differentiation and disrupted branching morphogenesis [25]. Additionally, Sox17 has been found to have a potential to reinitiate multipotent progenitor cell behavior, and it could activate the cell cycle in mature lung cells by inducing cell proliferation and the expression of the progenitor cell marker Sca-1 and cell cycle gene Cycling D1, as well as reducing the expression of TGF-beta1 responsive inhibitors, p15, p21 and p57, while inhibiting TGF-beta1 and Smad3 transcriptional activity [25].

## 4. *Sox* Genes and Lung Cancer

### 4.1. *Sox* Genes and Cancers

Improper regulation of *Sox* genes have been demonstrated to be associated with cancerous development of various types of cancer [20], such as gastric cancer [41,42], brain tumors [43,44], ovarian cancer [45], adenocarcinoma [46–49], hepatocarcinoma [50], breast cancer [51–53], melanomas [54], prostate cancer [55–58], colon carcinoma [59], and the lung cancer [18,36,60–69]. The role of the *Sox* gene family in the cancerogenesis has been attributed to their properties involving in the regulation of cell differentiation, proliferation, and survival in multiple essential processes. Although most *Sox* genes show a property of oncogenes in many types of cancers, different members of the *Sox* gene family may play distinct roles in various types of cancers; some of them show an oncogenic potential to promote the development of cancers [70], whereas others may behave as a tumor suppressor gene to block the growth of carcinomas [71,72]. For instance, Sox9 plays broadly roles in cancerogenesis and is overexpressed in many types of human cancers, where Sox9 exhibits pro-oncogenic properties of promoting cell proliferation, inhibiting cell senescence, and collaborating with other oncogenes in neoplastic transformation [73]. The expression of Sox9 was upregulated in hepatocellular carcinoma and lung adenocarcinoma, and it was associated with tumor progression and poor prognosis of the diseases [46,50]. On the other hand, a downregulated expression of Sox7 in lung adenocarcinoma was a poor prognosis marker for patients with this type of cancer [47]. Interestingly, the identical *Sox* gene exhibits a dual functional role in cancers, which may play opposite functions in different cancer types. For example, Sox2 was overexpressed in glioblastoma multiform as compared to normal cell lines. Such ectopic expression was sufficient to induce invasion and migration of glioma cells, suggesting that it contributed to tumor development in this study [74]. In contrast to the characteristic of oncogene, Sox2 was capable of inducing cell-cycle arrest and apoptosis in gastric epithelial cells, and the loss of Sox2 expression might be relevant to gastric cancerogenesis and poor prognosis [41]. Similarly, an upregulation of *Sox9* gene was capable of inhibiting the growth of melanomas [54]. In glioma cells, however, a downregulation of Sox9 expression could inhibit the cell growth, induce the cell arrest and enhance the apoptosis [70]. Furthermore, *Sox* genes might express in malignant cells to specifically stimulate the immune system in order to produce their relevant antibodies. This could be utilized as an index for diagnosis and therapy for cancers [64]. Table 1 lists the *Sox* genes related to lung cancer. Similarly seen in other types of cancer, the role of *Sox* genes and proteins have also been demonstrated that they play roles in the oncogenesis, tumor suppression, and cancer stem cells’ (CSCs) maintenance in lung cancer. More important, the same *Sox* gene shows distinct functions in diverse types of lung cancer, indicating that different types of lung cancer possess specific cell contexts [68,69,75,76].

### 4.2. Oncogenic Potential of *Sox* Genes in Lung Cancer

A number of studies have shown strong expression of Sox2, Sox4, and Sox11 in most SCLCs [61]. This implied the potential roles for the Sox transcriptional factors in this type of lung cancer. Among them, the role of *Sox2* gene in lung cancer is the one best recognized, which is relevant to aggressive tumor behavior, poor outcome, and/or the increasing risk of recurrence [48,62,76,80]. Sox2 was found to be a potential cell-lineage gene highly expressed in both of the human SCLC [66,80] and NSCLC [63,75,83]. Previous studies in mice revealed that the high level of Sox2 expression in lung epithelial cells was favorable to promote lung cancerogenesis [80]. Such findings were supported by the studies in patients with lung cancer; high expression of Sox2 was detected in lung SCC and stage I lung cancer of these patients, in which the Sox2 was found to be in concert with p63 to influence the tumor differentiation [48,83]. Additionally, autoantibodies against Sox2 were also detected in sera from SCLC patients [48,80,83,84]. The potential role of *Sox2* gene in lung cancer was further supported by a recent finding of identification of *Sox2* as a frequently amplified gene [66]. In this study, Rudin and colleagues performed a comprehensive genomic analysis to identify new recurrent somatic mutations in SCLC; twenty-two significantly mutated genes were identified, including several members of *Sox* family of genes (*Sox3*, *Sox4*, *Sox5*, *Sox6*, *Sox9*, *Sox11*, *Sox14* and *Sox17*) in SCLC. In addition, *Sox2* amplification was found in approximately 27% of the SCLC samples determined by immunohistochemical staining (IHC) or fluorescent *in situ* hybridization (FISH) assays. Moreover, Sox2 expression inhibited by shRNAs led to a decreased proliferation of *Sox2*-amplified SCLC cells. These results suggest that *Sox2* gene may be a potential target for therapeutic intervention in SCLC.

Aside from the Sox2, the expression of Sox4 was also elevated in several types of cancer [85,86], in which lung cancer had the greatest levels of Sox4 expression [61,65]. Through *in vitro* knocking out the expression of Sox4, the induction of apoptosis and growth suppression in cancer cells were demonstrated [55,87,88]. These studies clearly indicated a correlation of *Sox4* with lung cancer. The relationship of *Sox4* to cancerogenesis of lung cancer was further attested by a polymorphic study in patients with NSCLC. In this study, a gradual increased mutation rate with the pathological stages of NSCLC were observed, suggesting that *Sox4* gene mutation was correlated with the pathological stages of this type of cancer, even though no association of *Sox4* gene mutation with pathology histological types of tumor was determined [18].

Other than the *Sox2* and *Sox4*, *Sox9* and *Sox18* also showed oncogenetic properties in lung cancer. Recent studies validated that Sox9 was capable of promoting lung AC proliferation potential through the regulation of the expressions of p21 and CDK4. This suggested that Sox9 was a new hallmark of lung AC, in which Sox9 might contribute to the gain of tumor growth potential [46]. The aberrant methylation in the promoter of *Sox18* gene in lung cancer also suggested that inactivation of Sox18 expression might play a crucial role in the malignance [89]. Four Sox family members (Sox2, Sox4, Sox9, and Sox11) are currently determined with high levels of expression in various types of lung cancers, but the patterns of their expression among the lung cancer types are strongly different, particularly in NSCLC, in which increased levels of Sox4 expression is preferentially in ACs. On the other hand, high levels of Sox2 and Sox9 are more commonly seen in the SCC type [61].

Apart from their oncogenic properties, Sox transcriptional factors also exert inhibitory functions in the development and progression in a variety of cancers. Presently, the tumor suppressor characteristics of Sox proteins in lung cancer are mainly focused on members of Sox group F (Sox7, Sox17, Sox18). A decreased expression of Sox7 determined by quantitative real-time reverse transcription polymerase chain reaction (qRT-PCR) and/or IHC in lung AC, has demonstrated to be associated with poor differentiation of cancer cells and a better overall survival rate. This suggests Sox7 may serve as a prognostic biomarker for lung AC [47].

Genetic mutations, as well as epigenetic modifications have been increasingly recognized to play major roles in the cancerogenic process. Table 2 lists some of genetic and epigenetic abnormalities of *Sox* genes and their clinical relevance in cancers, particularly in the lung cancer. In an epigenetic study, promoter methylation of *Sox17* promoter was found in 60.2% of primary human lung cancer specimens [36]. Owing to aberrant and heterogeneous methylation in their promoter CpG islands, both expressions of Sox17 and Sox18 were silenced in SCLC [36] and NSCLC [60]. In addition, colony formation assays further demonstrated that expression of Sox17 inhibited lung cancer cell proliferation and differentiation, in part through a suppression of Wnt signal pathway [36]. Moreover, in addition to a biomarker of poor prognosis in lung cancer, *Sox2* amplification and upregulation were frequent events in lung SCC, which were associated with indicators of favorable prognosis in this study [68].

## 5. *Sox* Gene and Lung Cancer Stem Cells

Cancer stem cells (CSCs) are defined as a small subset cells within tumors which possess the capacity of self-renewal, potential differentiation, and give rise to heterogeneous cancer cell types [91]. CSCs thereby have the ability of initiation, maintenance, and recurrence of a tumor [92]. The isolation and characterization of lung stem and progenitor cells are an important step towards the understanding of pathogenesis of lung disease, as well as the identification of the target cells of transformation in lung cancerogenesis [93]. A pioneering study conducted by Kim *et al.* has identified bronchioalveolar stem cells (BASCs) in the distal lung [94]. This subset of population of stem cells resided at the bronchioalveolar duct junction, with a capacity to repair bronchiolar and alveolar injury and proliferate during epithelial cell renewal *in vivo*. Importantly, BASCs had an ability of expansion in response to oncogenic Kras in culture and in precursors of lung cancers *in vivo*, which was indicative of the putative cells of origin for lung adenocarcinoma, and a direct evidence of the existence of lung cancer stem cells [94]. The adult lung stem cells and characterization of lung cancer stem cells have been extensively reviewed recently [93].

**Figure 1:**
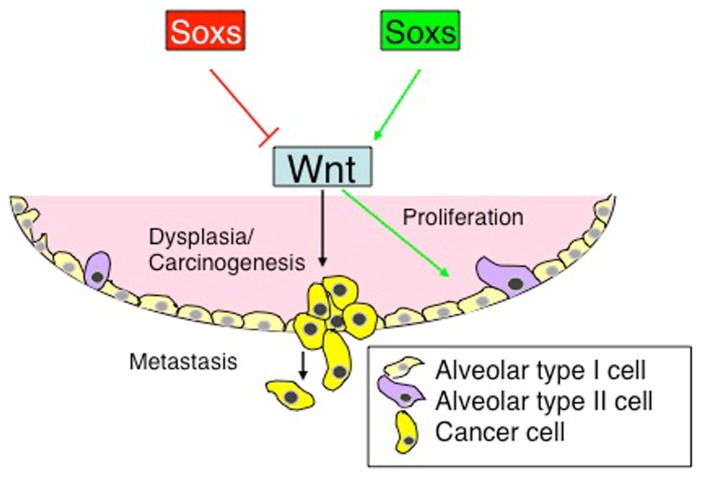
Potential roles of *Sox* genes in the development of lung cancer by a mechanism of the interaction of Sox and Wnt signaling. In the cancerogenesis of lung cancer, Sox proteins could play a role in the cancerogenesis of lung cancer by either repressing (such as Sox17) canonical Wnt signaling or enhancing (such as Sox4) the Wnt signaling, by which the Wnt/β-catenin signaling controls the determination of CSCs for proliferation, self-renewal and differentiation, and accordingly impacts the initiation, progression, and metastasis (EMT) of lung cancer through, in part, a mechanism of regulation of the expression of Wnt target genes, including those related to cell cycling, stem cell pluripotency, and EMT.

In addition to its pivotal role in the derivation and maintenance of embryonic stem cells (ES) and induced pluripotent stem cells (iPS), the Sox2 showed its potential to derive gastrointestinal CSCs [95,96]. As a stemness gene, *Sox2* plays an essential role in maintaining the stemness property of glioma-initiating cells (GICs), where the expression of *Sox2* gene could be induced by autocrine TGF-β through its target gene, *Sox4*[97]. The importance of Sox2 in maintaining lung CSC has also been investigated by Xiang and coworkers [69]. In their study, the upregulation of Sox2 and Oot4 in the side population (SP) cells of lung cancer led the cancer cells to shift to CSCs. On the other hand, knocking down Sox2 expression showed a reduced ability of migration and enhanced apoptosis in these cells [69]. The molecular mechanism of *Sox2* gene in promoting development of lung cancer stem cells and enhancing the potential of cancerogenesis might be related to the elevation of the expression of oncogenes c-Myc, Wnt1, Wnt2 and Notch1 [62]. Indeed, increased expression of either Sox2 or nuclear β-catenin was found to be associated with distant metastases of colon cancer [98]. In the lung, β-catenin was found to regulate basal progenitor cell fate and subsequent SCC progression by modulating epithelial–mesenchymal transition (EMT) [99]. Moreover, mutations in Wnt pathway components enable the leading of the constitutive signaling and have been linked to the cancerogenesis of many types of cancer [100,101]. Additionally, a recent work demonstrated that a combinatoral activation of both canonical Wnt signaling and Kras pathway showed a remarkable increase of lung cancer formation with a more progenitor-like phenotype [102,103]. Emerging evidence has also suggested a widespread role of *Sox* gene in modulating Wnt signaling in the development and in diseases, in which Sox proteins either repressed or activated Wnt signaling (Figure 1) [11]. It will be conceivable that a therapeutic strategy specifically targeting signaling molecules utilized by lung CSCs, such as the *Sox* gene, could be beneficial in treating a dreadful disease like lung cancer.

## 6. *Sox* genes for Diagnosis and Therapeutic Targets in Lung Cancer

Sox protein of members of the Sox B1 group (Sox1, Sox2, Sox3, Sox21) has been demonstrated to be highly immunogenic in patients with SCLC [78]. The autoantibody to these transcriptional factors was frequently and stably presented in the serum of patients, as compared with normal subjects. This suggests that Sox group B, except Sox14, may serve as serological markers for SCLC [67,78,79]. Moreover, aside from their diagnostic use, the determination of Sox group B1 antibodies was able to discriminate the difference between SCLC coupled with Lambert-Eaton myasthenic syndrome (LEMS) and nontumor LEMS, although it seemingly does not improve prognosis in SCLC [64,67]. Sox2 protein also was detected in NSCLC and SCC as a marker for diagnosis and therapeutic targets, even though it did not show a high sensitivity and specificity for SCC [62,63,104]. In addition, overexpression of Sox4 was detected in all types of lung cancer, including SCLC and NSCLC, and the antigen peptide derived from Sox4 was capable of activating CD4^+^ and CD8^+^ T cells recognizing Sox4 positive lung tumor cells, by which Sox4 might serve as a vaccine antigen candidate for the treatment of both SCLC and NSCLC [81]. These evidences clearly indicated that Sox proteins and autoantibodies might be effective molecular markers and/or therapeutic targets of lung cancer.

## 7. Concluding Remarks

The roles of Sox transcriptional factors in essential development and physiological processes have been rapidly elucidated since the discovery of their first member two decades ago. In this review, the potential role of *Sox* gene family members in lung cancer was emphasized by discussing current knowledge of the role of Sox transcriptional factors in lung development and lung cancer, as previously discussed. It is now unambiguous that the Sox family plays indispensable roles in lung morphogenesis, cancerogenesis, as well as lung CSC maintenance through a variety of mechanisms, such as epigenetic modification, interaction with Wnt signaling, and immune system modulation. It will be important to explore the association of the recently discovered mutations in *Sox* genes and their implication in varied subtypes of lung cancer. Moreover, the dual functional role of Sox family transcriptional factors in lung cancer implies a diverse function and mechanisms of *Sox* gene in cancerogenesis of lung cancer, which requires further investigation. Additionally, the specific expression of the Sox factor in lung cancer tissues and the autoantibody to Sox protein in the serum has provided a valuable target for diagnosis and the development of targeting agents for lung cancer. Of equal importance, the ability of Sox to induce antigen-specific T-cell response also offers a feasibility to develop vaccine-based immunotherapeutic strategies by which the comprehensive Sox-related anti-tumor immune response may be activated.

To date, however, only several *Sox* gene members have been unsystematically studied; it is a necessity to extensively investigate their roles in the essential development and diseases, as well as to further explore the roles of other *Sox* family members in this context, and the impact of genetic mutation and epigenetic modification of the *Sox* gene in lung cancer development. In spite of increasing evidence that suggests that *Sox* gene family members are correlated with the lung development and cancer formation at some extent, the underlying molecular mechanisms of *Sox* gene on lung morphogenesis and cancerogenesis have yet to be fully established.

## Figures and Tables

**Table 1:** Evidence and role of Sox family transcriptional factors in lung cancer.

*Sox* genes	Evidence or role in lung cancer
*Sox1*	Autoantibody to Sox1 detected in the serum of patients with SCLC [67,77–79].
*Sox2*	Autoimmunity found in patients with SCLC [64,67,78,79]; strongly expressed in both of NSCLC and SCLC tumor cells determined by IHC and/or FISH assays [62,63,66,76]; overexpression leads to lung cancer with poor prognostic outcome [48,80]; gene mutation(s) observed in SCLC [66]; inhibition of expression in cancer stem cells suppress the growth and metastasis of lung cancer [69].
*Sox3*	Autoantibody detected in patients with SCLC [67,78,79]; gene mutation(s) observed in SCLC [66].
*Sox4*	Autoantibody detected in the serum of patients with SCLC [81]; strong expression detected in SCLC tumor cells by IHC [61]; gene mutations found in SCLC and might be correlated with the lung cancerogenesis and tumor metastasis [18,65,66,82].
*Sox5*	Gene mutation (s) determined in SCLC [66]
*Sox6*	Gene mutation (s) observed in SCLC [66]
*Sox7*	Downregulation was correlated with a poor prognosis in patients with lung AC [47].
*Sox9*	Overexpression promoted lung adenocarcinoma cell proliferation [46]; gene mutation(s) detected in SCLC [66].
*Sox11*	Gene mutation (s) found in SCLC [66]
*Sox14*	Gene mutation (s) observed in SCLC [66]
*Sox17*	Gene mutation (s) detected in SCLC [66]
*Sox18*	Heterogeneous methylation was found in the promoter of gene [60].
*Sox21*	Autoantibody detected in the serum of patients with SCLC [67,78,79]

**Table 2:** Genetic and epigenetic abnormalities of *Sox* genes and their clinical relevance in cancers.

*Sox* gene	Abnormality or mutation	Potential clinical implication	Reference
*Sox2*	Gene amplification	Associated with the prognosis of SCLC and lung SCC	[61,66,68,74,84]
	Gene methylation	Associated with the progression of malignant gliobastoma and gastric cancer	[41,74]

*Sox3*	N161K mutation	Associated with the progression of SCLC	[66]

*Sox4*	Point mutations	Correlated with the stages of NSCLC	[18]
	Gene amplification	Associated with the prognosis of SCLC and NSCLC	[61,65]
	S395X mutation	Increase the ability of transform in cell line	[65]
	D398V mutation	Found in SCLC samples	[66]

*Sox5*	V316F, R547L mutations	Found in SCLC samples	[66]

*Sox6*	D494Y, G728V mutations	Found in SCLC samples	[66]

*Sox7*	Methylation of CpG islands near the gene promoter	Correlated with the poor prognosis of patients with myelodysplastic syndrome	[90]

*Sox9*	S64R mutation	Found in SCLC samples	[66]
	CpG methylation of promoter gene	Associated with progression of bladder cancer and gastric cancer	[42,72]

*Sox11*	Gene amplification	Associated with the prognosis of SCLC	[61]
	E101 * nonsense change	Found in SCLC samples	[66]

*Sox14*	M240I mutation	Found in SCLC samples	[66]

*Sox18*	Promoter gene CpG methylation	Found in NSCLC tissues	[60]

* nonsense mutation.
